# Morphological traits and microbiome diversity in the free-living nematodes *Acrobeles complexus* and *Zeldia punctata*

**DOI:** 10.1371/journal.pone.0341018

**Published:** 2026-01-30

**Authors:** Ebrahim Shokoohi, Peter Masoko

**Affiliations:** Department of Biochemistry, Microbiology, and Biotechnology, University of Limpopo, Private Bag X1106, Sovenga, South Africa; Chandigarh University, INDIA

## Abstract

Morphological adaptations play a key role in shaping the feeding behavior and microbiome associations of Cephalobidae nematodes. To investigate how morphology influences nematode-associated microbiomes, we selected two widely distributed species: *Acrobeles complexus*, exhibiting elaborated oral structures, and *Zeldia punctata*, with simpler oral morphology. Unlike earlier studies that reported the microbiomes of *A. complexus* and *Z. punctata* independently, this study is the first to directly compare the two species. By integrating *in silico* re-analysis of our previously published microbiome datasets with new light microscopy and scanning electron microscopy (SEM) observations, we demonstrate how morphological adaptations, such as labial probolae and cuticle structures, shape associated bacterial communities. Our results revealed that *A. complexus* harbored a more diverse bacterial community than *Z. punctata*. Morphology showed that the complex oral structures of *A. complexus* facilitated selective bacterial capture, supporting greater microbial diversity compared to the simpler morphology of *Z. punctata*. Although statistical significance was not observed, the two species showed distinct patterns of microbial richness and diversity. Principal Coordinate Analysis (PCoA) revealed clearly separated bacterial community structures between the species. Linear discriminant analysis effect size identified potential microbial biomarkers at the genus level, including Firmicutes and *Clostridium* in *A. complexus* and Actinobacteria and *Pseudomonas* in *Z. punctata*. Predicted functional pathway analysis revealed notable differences in microbial metabolism, such as enrichment of bacterial secretion systems in *A. complexus* and amoebiasis and lipid metabolism pathways in *Z. punctata*. This study highlights the role of morphological adaptations in shaping microbiome composition in Cephalobidae nematodes and provides insights into the contribution of free-living bacterivorous nematodes to soil microbial balance. These findings lay the groundwork for further studies on nematode-mediated microbial interactions in soil ecosystems.

## Introduction

Nematodes form an integral part of the soil biome, contributing to ecological processes such as decomposition, microbial regulation, and nutrient turnover, and they are found in diverse soil types and associated with many plant species [1]. Soil is home to two broad categories of nematodes: free-living nematodes and parasitic nematodes, the latter comprising both plant-parasitic and animal-parasitic groups. Plant-parasitic nematodes actively infest the roots and tissues of plants, often leading to reduced growth, nutrient deficiencies, and increased susceptibility to diseases [2]. In contrast, free-living nematodes play a beneficial role in the soil ecosystem by contributing to nutrient cycling, decomposing organic matter, and helping to control populations of harmful microorganisms [3]. Each of these groups has a unique impact on plant health and soil dynamics, highlighting the importance of understanding their roles in agricultural and natural environments.

Free-living bacterivorous nematodes are the primary group of nematodes that feed on bacteria in the soil [4]. Members of the Cephalobidae family, including *Acrobeles* and *Zeldia*, are widely distributed bacterivorous nematodes commonly encountered in soil environments [3,5]. Numerous studies have demonstrated that nematodes serve as effective indicators of soil health [6–8]. Among these microscopic organisms, bacterivorous nematodes stand out due to their widespread distribution compared to other trophic groups. This notable presence underscores their significant role in assessing soil conditions, making them invaluable bioindicators in environmental monitoring and ecological research. Their abundance can provide crucial insights into the microbial activity and overall health of the soil ecosystem. Free-living nematode microbiomes have been previously studied [9–10], showing the diversity of bacteria associated with free-living nematodes.

Despite the growing body of research on various soil organisms, a comparative analysis of the microbiome associated with free-living nematodes has yet to be undertaken. Investigating this aspect could provide valuable insights into the specific bacterial communities that interact with these nematodes, offering a better understanding of their roles in soil ecosystems and their relationships with different host organisms. Additionally, this knowledge could further elucidate the complex interactions within the soil microbiome and its impact on soil health and fertility.

Although free-living nematodes inhabit soil environments rich in microbial diversity, the bacterial taxa analyzed in this study were not derived from bulk soil samples. Instead, all microbiome profiles originated from whole nematodes that were individually isolated and subjected to surface-sterilization prior to DNA extraction, as described in the original datasets. This procedure removes loosely attached external bacteria, ensuring that the bacterial sequences recovered represent only those that are ingested, internal, or tightly bound to the nematode cuticle. Therefore, the microbial communities reported here reflect the nematode holobiont rather than the surrounding soil, allowing for accurate species-specific comparisons between *A. complexus* and *Z. punctata* [11,12].

While we previously reported the microbiomes of *A. complexus* [11] and *Z. punctata* [12] independently, this study provides the first comparative analysis of the two species, linking their microbiomes to morphological adaptations documented by new SEM data.

The aim of this study was twofold: (1) to compare the microbiomes of *Acrobeles* and *Zeldia*, two common Cephalobidae nematodes, and (2) to investigate how morphological features influence their microbiome composition using scanning electron microscopy (SEM). The microbiome datasets, generated in our previous work [11,12], were retrieved from NCBI and subjected to *in silico* re-analysis using QIIME2 and LEfSe. Complementing this, SEM and detailed morphological observations—including labial probolae, cuticle, and oral structures—were conducted experimentally as part of this study, enabling a direct assessment of how nematode anatomy shapes associated bacterial communities.

## Materials and methods

### Study design

This study combined *in silico* re-analysis of publicly available microbiome datasets with original morphological observations. Microbiome composition of *A. complexus* and *Z. punctata* was assessed using previously published sequencing data, while scanning electron microscopy (SEM) was performed on nematode specimens collected for this study to examine labial probolae, cuticle, and oral structures. No governmental permits were required for this study because soil and plant-root sampling were conducted in non-protected areas at Dalmada and Sovenga Hills, Limpopo Province, South Africa. These locations do not fall within conservation areas or land under restricted environmental regulation. Field site access and permission to collect soil and root samples were granted by the respective local landowners and community authorities. All sampling activities complied with South African environmental research guidelines and standard ethical procedures for non-endangered plant material.

### Origin and definition of nematode-associated bacteria

To clarify the origin of the bacteria included in this study, we emphasize that the microbiome datasets were generated from whole nematode specimens that underwent surface-sterilization prior to DNA extraction. Individual nematodes were washed in 1% sodium hypochlorite for three minutes, followed by a three-minute immersion in Salvon antiseptic solution (Johnson & Johnson, South Africa), and subsequently rinsed three times in sterile distilled water to remove loosely attached external bacteria. After surface-sterilization, total genomic DNA was extracted from the entire nematode body. Therefore, the bacterial taxa detected in this study may represent: (i) bacteria present within the digestive tract; (ii) bacteria tightly attached to the cuticle that were resistant to sterilization; and (iii) bacteria ingested during natural feeding activity.

Throughout this manuscript, the term **“**nematode-associated bacteria**”** is used in this ecological context and does not imply obligate or symbiotic endosymbiosis. Instead, it refers to all bacterial taxa recovered from the nematode holobiont after sterilization, consistent with established practices in free-living bacterivorous nematode microbiome research.

### Microscopy imaging (Scanning Electron Microscopy and Light Microscopy)

The specimens of *A. complexus* and *Z. punctata* were obtained from soil samples that were collected from tomato and maize fields, respectively, situated in Limpopo Province, South Africa. After their examination and identification, a few specimens preserved in glycerine were re-processed to their observation under SEM following the protocol by Shokoohi [13]. The nematodes were hydrated in distilled water, dehydrated in a graded ethanol and acetone series, coated with gold, and observed with a Zeiss Sigma 500VP–Field Emission Scanning Microscope (FESEM) with Gemini column.

Light microscopy was used to document key diagnostic structures, particularly the lip region and associated feeding adaptations. Images were captured using a VWR compound microscope (VWR International, Milan, Italy) fitted with a Moticam A16 digital camera. Bright-field illumination was used to obtain LM images of the labial region. All procedures followed standard nematode morphological examination protocols suitable for comparative analyses.

### Nematode phylogenetic analysis

For phylogenetic analysis, *Teratolobus* sp. (KJ652552) was selected as an outgroup. The ribosomal DNA sequences were analyzed and edited with BioEdit [14] and aligned using CLUSTAL W [15]. The GTR + G model was selected using jModeltest 2.1.10 [16,17], and then initiated with a random starting tree and ran with the Markov Chain Monte Carlo (MCMC) for 10^6^ generations [18]. The sequences *A. complexus* (OR889721, OR889722, and OR889724) and *Z. punctata* (MZ254892) were used for phylogenetic analysis in the present study.

### Nematode microbiome analyses

No soil samples were sequenced for microbial composition. All bacterial taxa reported in this study originated from DNA extracted directly from surface-sterilized nematodes. This study involved an *in silico* re-analysis of publicly available microbiome datasets deposited in NCBI for *A. complexus* [11] and *Z. punctata* [12]. Nematode microbiome sequences were retrieved from NCBI, including SAMN39413027, SAMN39413028, and SAMN39413029 for *A. complexus* [11], and SAMN20693819, SAMN20694755, SAMN20694756, and SAMN20694757 for *Z. punctata* [12]. Microbiome bioinformatics analyses were performed using QIIME 2 (version 2017.4) [19–21]. Raw sequence data were quality filtered using DADA2 via q2-dada2 [22], and all amplicon sequence variants (ASVs) were aligned with MAFFT through q2-alignment [23]. Phylogenetic trees were constructed using FastTree2 via q2-phylogeny [24]. Samples were rarefied to 4,235 sequences per sample (subsampled without replacement) [25], after which alpha-diversity metrics—including observed features and Faith’s phylogenetic diversity—were calculated using q2-diversity [26]. Taxonomic assignment of ASVs was performed with q2-feature-classifier using the Greengenes 13_8 99% OTUs reference database [19,20,27].

Alpha diversity, which measures diversity within samples, is assessed using the evenness, as well as the Shannon indices for both samples. The values of these indices are associated with the number of species and the evenness of their distribution. Beta diversity, which indicates diversity between samples, is evaluated using the Bray-Curtis and Jaccard indices. The distance represented in their graphical outputs reflects the differences between samples. Principal Coordinate Analysis (PCoA) is conducted using relative abundance data [28]. The Linear Discriminant Effect Size (LEfSe) method identifies differences in relative abundance between two groups, utilizing an alpha value of 0.05 and a logarithmic score (LDA) threshold of 2. The bacteria displayed have LDAs ranging from −2.0 to 2.0 and are colour-coded in green and red to indicate enrichment or reduction compared to the other group [29]. To explore the differences in microbial composition between the two genera, Venn analysis was performed based on the genera’s profile using Venny 2.1.0 [30].

### Ethical considerations

This study did not involve human participants, human data, or vertebrate animals; therefore, ethical approval and informed consent were not required.

## Results

### Molecular confirmation of nematode species

The identity of the South African specimens of *A. complexus* and *Z. punctata* was confirmed using 28S rDNA sequences. Sequence comparison with previously published data showed 100% identity with reference sequences of each species, consistent with morphological identification. These results confirm that the specimens examined in this study correspond to the expected species, validating their use in subsequent microbiome and morphological analyses (S1 Fig).

### Composition of the bacterial community

**Fig 1:**
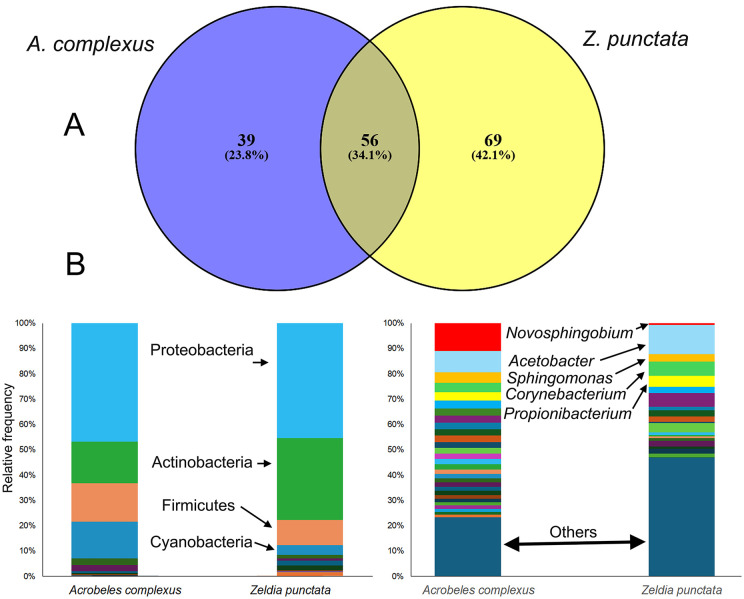
Venn diagrams and sample microbiome analysis of *A. complexus* and *Z. punctata* (A). Relative frequency of the microbial population at the phylum and genus level for *A. complexus* and *Z. punctata* **(B)**.

These results are based on the *in silico* re-analysis of previously published microbiome datasets obtained from NCBI. After removing noise and aligning the data, we were able to assemble 518 sequences across the seven nematode samples that were biologically replicated, including four samples belonging to *Z. punctata* and three samples belonging to *A. complexus*. These sequences represented 164 ASVs at the genus level. Among these ASVs, we found 95 for *A. complexus*, and 125 ASVs for *Z. punctata*. According to the result (Fig 1A), *A. complexus* and *Z. punctata* share 56 ASVs. In contrast, *A. complexus* had 39 and *Z. punctata* 69 unique ASVs at the genus level.

The results showed a thorough examination of the taxonomic affiliation of the representative sequences, which indicated certain types of bacteria found in association with *A. complexus* and *Z. punctata* (Fig 1B). Specifically, the most abundant bacteria belong to the phyla Proteobacteria, Actinobacteria, Firmicutes, and Cyanobacteria, as documented in Fig 1B. These phyla were signified by the bacterial classes Alphaproteobacteria, Actinobacteria, and Clostridia (S2 Fig). Subsequently, signified by the bacterial orders Sphingomonadales, Clostridiales, Actinomycetales, Rhizobiales, and Rhodospirillales (S3 Fig). The most dominant families were Sphingomonadaceae, Acetobacteraceae, and Ruminococcaceae (S4 Fig). The most dominant genera were *Novosphingobium*, *Acetobacter*, *Sphingomonas*, *Corynebacterium*, and *Propionibacterium* (Fig 1B).

### Bacterial diversity of *A. complexus* and *Z. punctata*

**Fig 2:**
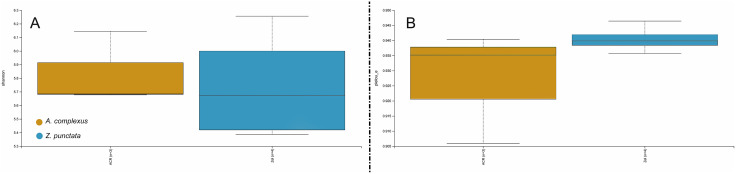
Alpha diversity analysis of microbiome associated with *A. complexus* and *Z. punctata*, including Shannon (A) and evenness diversity index (B).

Alpha diversity reflects the Shannon and evenness of microbiota in *A. complexus* and *Z. punctata*. The results are shown in the box plot of Fig 2. The results showed that the Shannon indices (Fig 2A) in *A. complexus* and *Z. punctata* were not significantly different (Kruskal–Wallis test; p-value = 0.723674). The same scenario was observed with evenness (Fig 2B), indicating that there were no significant differences among the populations of *A. complexus* and *Z. punctata* (Kruskal–Wallis test; p-value = 0. 157299). However, all alpha diversity indices (including Chao1) showed no significant differences between *A. complexus* and *Z. punctata* population groups, suggesting that the species of the Cephalobidae had no direct influence on the diversity of the microbiome (Fig 2).

**Fig 3:**
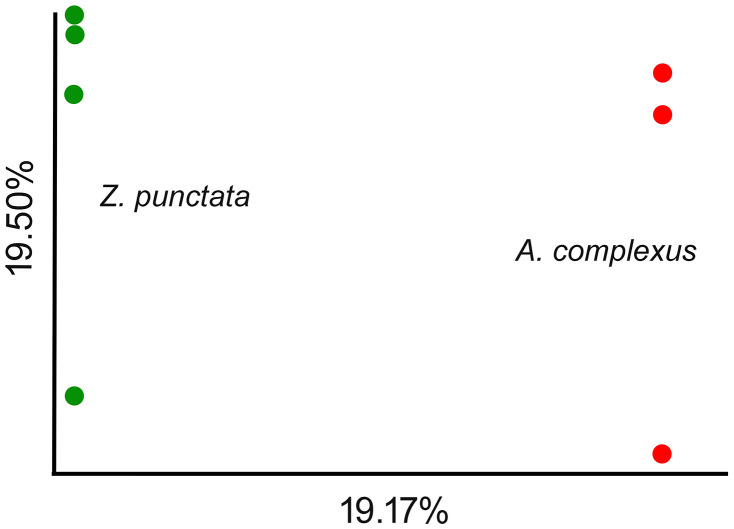
PCoA analysis of microbiome associated with *A. complexus* and *Z. punctata.*

Beta diversity, which reflects the similarity of microbiome associated with different species, is shown in Fig 3. The result indicated that two axes explained 19.17% and 19.50% of the variability among two groups, including *A. complexus* (red circles) and *Z. punctata* (green circles). Despite the variance among the microbiome of the two groups, significant differences (p > 0.05) were not observed.

### Linear discriminant analysis of microbiome

**Fig 4:**
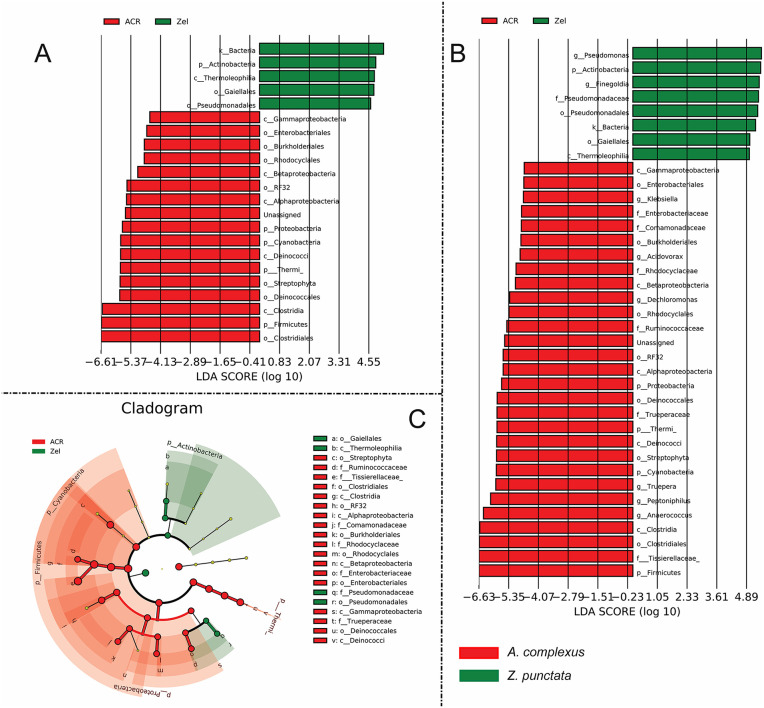
LDA scores display the distinct bacterial differences between *A. complexus* and *Z. punctata* (A, B). LDA scores > 2.0 were considered statistically significant. Cladogram revealing the phylogenetic distribution of the bacterial community associated with *A. complexus* and *Z. punctata* **(C)**. Differential abundance between categories was evaluated based on the factorial Kruskal–Wallis (KW) test and the pairwise Wilcoxon test (p < 0.05 and LDA score/effect-size Threshold = 2).

We used linear discriminant analysis (LDA) and effect size measurements to identify the different species in two or more nematode communities. The results of LDA analysis for the populations belonging to *A. complexus* and *Z. punctata* revealed significant differences (Kruskal–Wallis test; p-value < 0.05). Differential abundance analysis showed that bacterial phyla Actinobacteria was significantly (p-value < 0.05) associated with *Z. punctata*. In contrast, Proteobacteria, Cyanobacteria and Firmicutes were significantly (p-value < 0.05) enriched in *A. complexus* (Fig 4A, C). In addition, the LDA result showed that *Pseudomonas* and *Finegondia* genera were enriched in *Z. punctata* (Fig 4B). Whereas genera including *Anaerococcus*, *Peptoniphilus*, *Truepera*, *Dechloromonas*, *Acidovorax*, and *Klebsiella* were enriched in *A. complexus* (Fig 4B).

### Morphological traits studied for microbiome association

**Fig 5:**
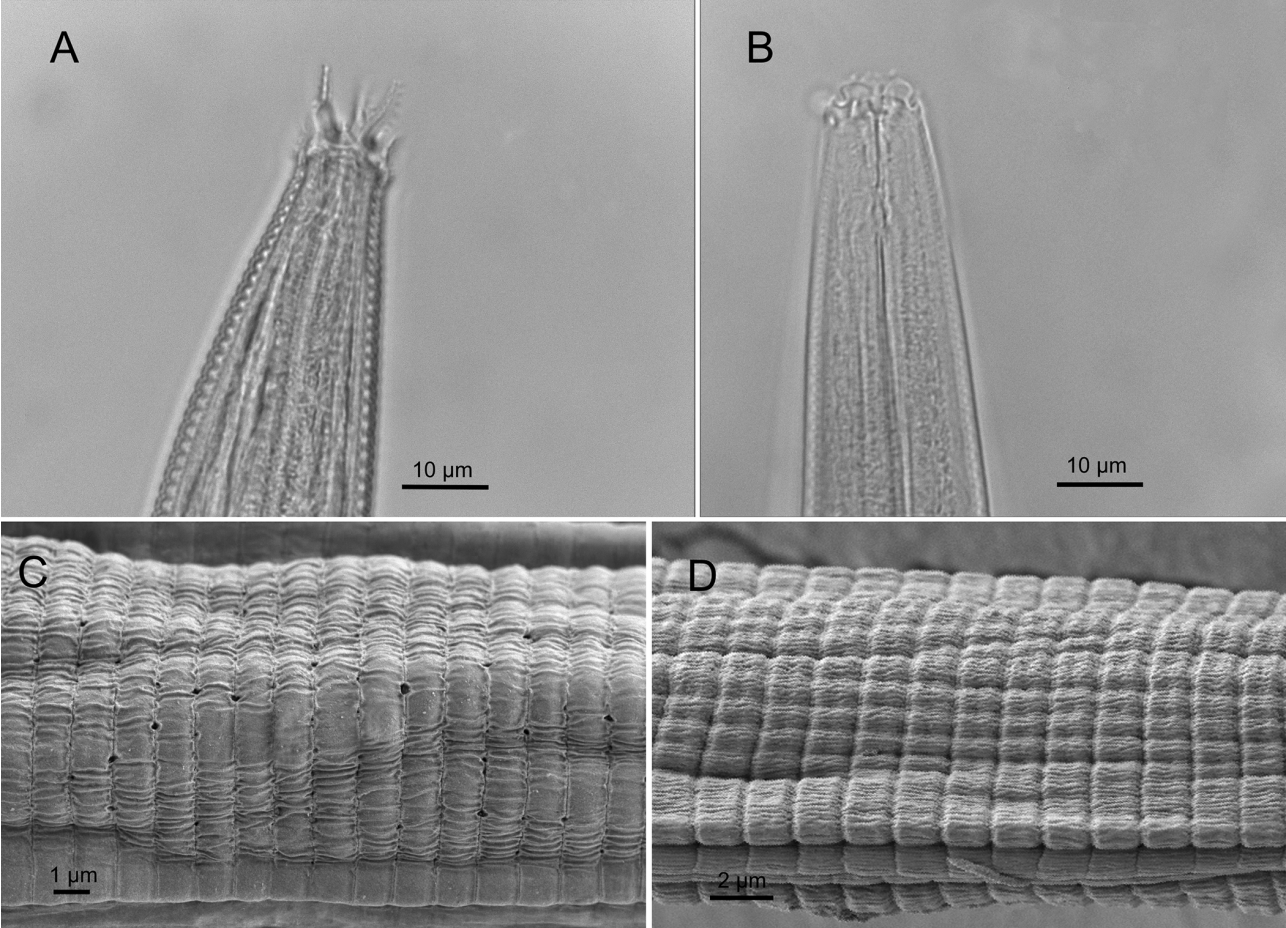
Morphological structures of *A. complexus* (A, C), and *Z. punctata* (B, D) used to interpret microbiome–feeding interactions. Scanning electron micrographs (SEM; C, D) showing the cuticle surface of the two nematode genera included in this study, alongside corresponding light-microscopy (LM; A, B) images of the lip region. *Acrobeles* exhibits elaborated labial probolae, whereas *Zeldia* displays reduced/low labial probolae. These structural differences are presented solely to demonstrate the morphological basis underlying the study’s objective, assessing how variation in the lip apparatus may influence bacterial filtering and ingestion, and thereby shape the associated microbiome. No taxonomic revision or formal species description is intended.

The following observations were obtained experimentally using light and scanning electron microscopy (SEM) on specimens collected for this study. The samples of *A. complexus* and *Z. punctata* were analyzed using LM and SEM to determine the relationship and effect of the labial probolae and cuticle on the microbiome, which attaches to the body or goes through inside the internal organs of nematodes. Light microscopy revealed that the labial probolae of *A. complexus* (Fig 5A) are well developed, bearing approximately 5–7 distinct tines with an apical prong. Cephalic probolae were also visible, each consisting of 5–6 membrane-like tines on either side. In contrast, the labial probolae of *Z. punctata* were markedly reduced and appeared as a low, smooth structure without tine (Fig 5B). SEM observations of the cuticle showed that *A. complexus* possesses a regularly annulated cuticle, with occasional small pores visible on the surface (Fig 5C). In contrast, the cuticle of *Z. punctata* appeared slightly tessellated in the mid-body region and lacked visible pores (Fig 5D).

## Discussion

Because each nematode was individually isolated, identified, and surface-sterilized prior to DNA extraction, the bacterial taxa recovered can be reliably attributed to the specific nematode specimen rather than the surrounding soil or other co-occurring species. While our earlier publications focused on single-species microbiome profiles [11,12], this study extends those findings by offering a comparative perspective and integrating SEM observations. This combined approach highlights how differences in oral and cuticular structures can influence the bacterial associations of *A. complexus* and *Z. punctata*. The family Cephalobidae comprises free-living bacterivorous nematodes that are common in soil ecosystems and play a role in nutrient turnover and soil structure [31]. Both *A. complexus* and *Z. punctata* occur in light soils across several regions, including South Africa [11,12].

In this study, the microbiome associated with *A. complexus* consisted mainly of Proteobacteria, Firmicutes, and Cyanobacteria. The relatively high abundance of Proteobacteria aligns with findings from agricultural soils where long-term land-use change, fertilizer input, and manure application favor members of this phylum [32,33]. Because *A. complexus* was collected from a tomato field under intensive cultivation, the bacterial community associated with this nematode likely reflects these soil conditions. Firmicutes, including spore-forming genera such as *Bacillus*, have important ecological and industrial roles and can promote plant growth [34–36]. Cyanobacteria, represented by genera such as *Nostoc*, may contribute to bioremediation or biocontrol functions in soil [37]. These three phyla were present in both nematode species but occurred at higher relative abundances in *A. complexus*. In contrast, Actinobacteria—one of the most diverse bacterial groups found across ecological niches—were more dominant in the microbiome of *Z. punctata* [38].

Despite sharing several bacterial phyla, each nematode species harbored unique genera. Four genera—*Acidovorax*, *Dechloromonas*, *Peptoniphilus*, and *Truepera*—were detected only in association with *A. complexus*. Members of *Acidovorax* include both plant pathogens and beneficial soil bacteria involved in iron mineral formation [39–41], and their presence may reflect the agricultural environment where *A. complexus* was sampled. *Dechloromonas* spp. are key denitrifiers in wastewater treatment and nutrient-rich soils, contributing to nitrogen cycling and soil fertility [42]. *Peptoniphilus* includes species known primarily from human-associated environments [43], whereas *Truepera* is known for its extremophilic physiology and metabolic versatility [44]. In contrast, *Pseudomonas* was the most notable genus uniquely associated with *Z. punctata*. This genus is widely recognized for its ability to enhance plant resilience under abiotic stress through the production of bioactive compounds that modulate plant hormone pathways [45]. The association of *Z. punctata* with *Pseudomonas* may relate to its collection from maize rhizosphere soil in a semi-arid environment, where such bacteria are ecologically important.

Overall, *A. complexus* displayed higher bacterial diversity, whereas *Z. punctata* contained a greater total number of bacterial taxa. Although both species were surface-sterilized using sodium hypochlorite followed by Salvon antiseptic treatment, some superficial bacteria may have remained, and SEM was used to interpret potential structural drivers of microbial retention.

Two key morphological features appear to influence microbiome composition in Cephalobidae: the labial probolae and the cuticle. Labial probolae are distinctive filtering structures that regulate bacterial entry into the nematode alimentary system [46]. In *A. complexus*, these probolae possess tines and elongated arms that likely enhance selective filtration and contribute to the broader bacterial diversity observed. In contrast, *Z. punctata* exhibits low, simplified probolae without tines, suggesting reduced filtering ability. SEM images also revealed that *Z. punctata* has a wider oral opening, which may facilitate entry of larger quantities of bacteria regardless of type. Cuticular structures may further affect bacterial interactions: *A. complexus* possesses fine pores that may serve as microhabitats for certain taxa [47], while *Z. punctata* has a tessellated cuticle that may trap bacteria on its surface.

### Comparative microbiomes with other free-living nematodes

The microbiomes of *A. complexus* and *Z. punctata* provide an opportunity to compare South African cephalobids with other free-living nematodes. For *Paracephalobus maximus* (= *Acrobeloides maximus*) [48], Baquiran et al. [49] reported enrichment of *Ochrobactrum*, *Pedobacter*, and *Chitinophaga*, whereas our South African species were dominated by Proteobacteria, Actinobacteria, Firmicutes, and Cyanobacteria, with genera such as *Novosphingobium*, *Acetobacter*, *Sphingomonas*, *Corynebacterium*, and *Propionibacterium*. Classical cephalobid-associated genera (e.g., *Pedobacter*, *Chitinophaga*) were not dominant in our samples, likely reflecting geographical, soil, or host-specific differences. Comparisons with *Caenorhabditis elegans* further suggest that environmental acquisition is a major determinant of microbiome structure in free-living nematodes. Although alpha-diversity metrics did not differ significantly between *A. complexus* and *Z. punctata*, both species showed moderate diversity levels comparable to culture-based *C. elegans* datasets. The distinct enrichment of Actinobacteria and Firmicutes in *A. complexus* and the presence of *Pseudomonas* and Finegoldia in *Z. punctata* likely reflect differences in feeding structures and soil environments.

### Alignment with morphological findings

SEM observations from this study showed that the complex, multi-tined probolae of *A. complexus* and the simplified probolae of *Z. punctata* correspond to the microbiome differences detected. Across free-living nematodes—including *P. maximus*, *C. elegans*, and other cephalobids—Proteobacteria and Bacteroidetes typically dominate, but species-specific signatures often correlate with feeding morphology, soil conditions, and biogeographical context. Thus, the microbiomes of South African *A. complexus* and *Z. punctata* reflect both shared cephalobid traits and distinct ecological adaptations.

In contrast, relatively consistent bacterial associations observed in some meiofaunal groups such as tardigrades, whose microbiome studies have frequently detected genera like *Brachybacterium*, *Pseudomonas*, *Methylobacterium/Methylorubrum*, *Lactococcus*, *Legionella*, *Polynucleobacter*, and members of Comamonadaceae as recurrent components of their microbial signal [50], our analysis of *A. complexus* and *Z. punctata* reveals a more selective pattern linked to feeding morphology. While many of the bacteria detected in tardigrades are now known to originate from food sources, culture media, or reagent contamination rather than representing true symbionts, the nematode-associated microbiome in our study appears more strongly shaped by structural features such as the labial probolae and stomatal filtering apparatus. This contrast highlights how different meiofaunal organisms—despite living in microbe-rich environments—can show fundamentally different modes of microbial association, with tardigrades generally lacking stable or specialized microbiota, whereas feeding-structure-driven filtering in rhabditid nematodes may allow more selective bacterial retention.

A limitation of this study is that the microbiome data were obtained from previously published datasets and analyzed *in silico*, whereas the SEM and LM observations were generated experimentally. In addition, the two nematode species were collected from different agricultural fields, which may have influenced the bacterial communities available to them and therefore shaped their detected microbiomes. These factors limit the extent to which environmental and host-specific effects can be separated. Nevertheless, the present results reveal consistent patterns linking nematode morphological features—particularly labial probolae and cuticle characteristics—to differences in microbiome composition. Future studies incorporating larger sample sizes, matched bulk-soil microbiome datasets, and controlled sampling conditions will be important for validating and extending these findings.

## Conclusion

This study demonstrates that morphological features—including labial probolae and cuticle patterns—significantly influence microbiome composition in Cephalobidae nematodes. By combining *in silico* re-analysis of previously published microbiome datasets with new morphological observations, we identified distinctive bacterial associations such as *Acidovorax* and *Dechloromonas* in *A. complexus* and *Pseudomonas* in *Z. punctata*. These findings highlight the potential of these nematodes as bioindicators of soil bacterial communities and ecosystem health. Although interpretation should consider limitations related to sampling environments and dataset size, future studies incorporating controlled conditions, additional species, and experimental verification will further clarify the ecological relationship between nematode morphology and microbiome diversity.

## Supporting information

S1 Fig Phylogenetic position of *Acrobeles complexus* and *Zeldia punctata* based on 28S rDNA sequences generated in the present study. (DOCX)

S2 Fig Relative frequency of the dominant bacterial classes associated with *Acrobeles complexus* and *Zeldia punctata.* (DOCX)

S3 Fig Relative frequency of the dominant bacterial orders associated with *Acrobeles complexus* and *Zeldia punctata.* (DOCX)

S4 Fig Relative frequency of the dominant bacterial families associated with *Acrobeles complexus* and *Zeldia punctata.* (DOCX)
