# Supplementary material for: Morphological traits and microbiome diversity in the free-living nematodes Acrobeles complexus and Zeldia punctata
Source: PLoS One. 2026 Jan 30;21(1):e0341018. doi: 10.1371/journal.pone.0341018 (PMC12857960; doi:10.1371/journal.pone.0341018)
Supplement: S2 Fig — (DOCX) [file pone.0341018.s002.docx]

**Morphological Traits and Microbiome Diversity in the Free-Living Nematodes *Acrobeles complexus* and *Zeldia punctata***

**Ebrahim Shokoohi^1^,** **and Peter Masoko^1^**

*^1^Department of Biochemistry, Microbiology, and Biotechnology, University of Limpopo, Private Bag X1106, Sovenga, 0727, South Africa.*

Corresponding author e-mail: Ebrahim.shokoohi@ul.ac.za

Supplementary files


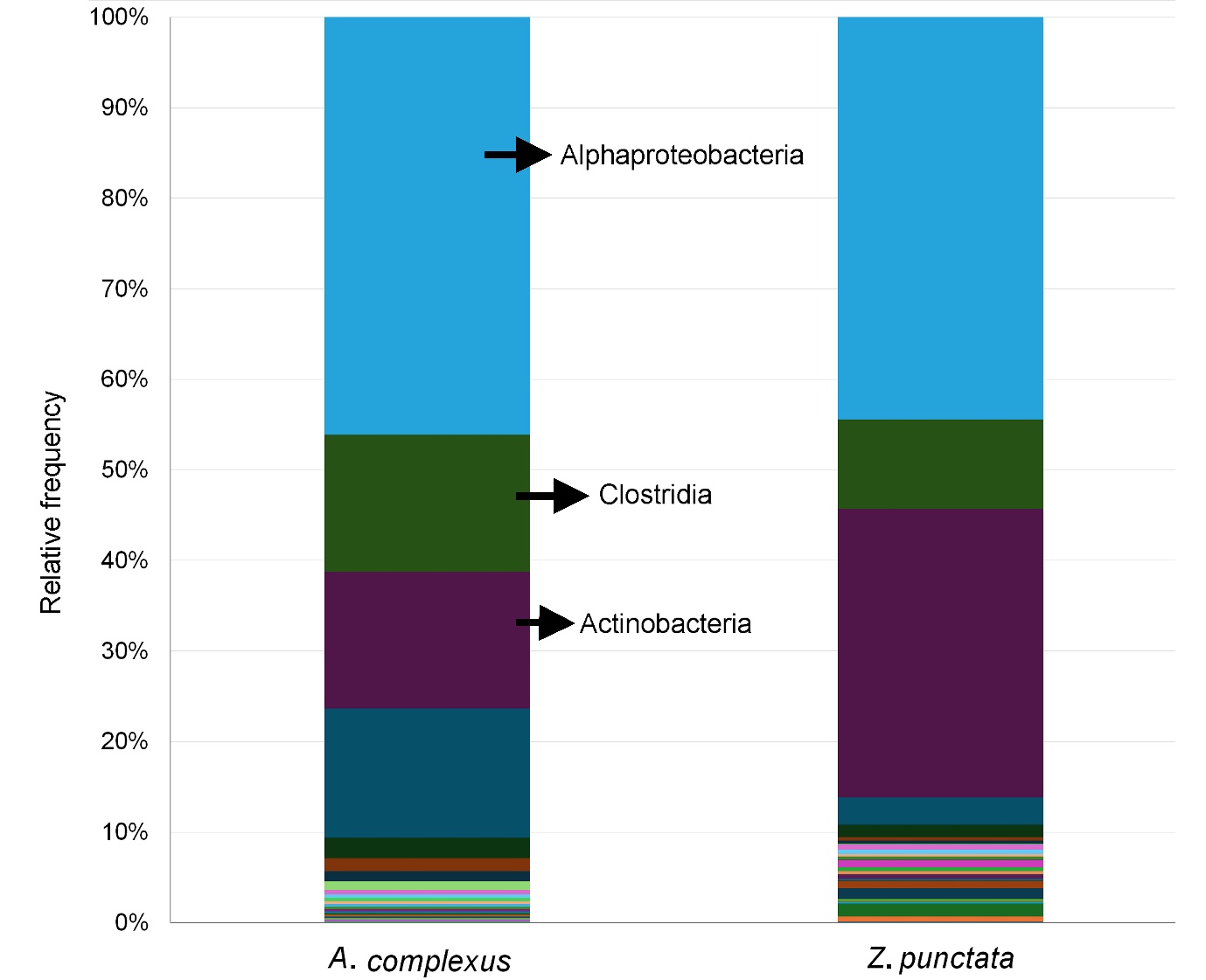


**S2 Fig.** Relative frequency of the dominant bacterial classes associated with *Acrobeles* *complexus* and *Zeldia* *punctata*.
